# Engineered Tan-CDs@AS-IV Nanosystem Orchestrates Mitochondrial Biogenesis and Intercellular Transfer to Restore Endothelial Function via PGC-1α and Cx43 Signaling Pathways

**DOI:** 10.3390/nano16110698

**Published:** 2026-06-04

**Authors:** Haoran Wang, Xiaoyu Wang, Shuo Liu, Chunzhao Liu

**Affiliations:** State Key Laboratory of Bio-Fibers and Eco-Textiles, Institute of Biochemical Engineering, College of Materials Science and Engineering, Qingdao University, Qingdao 266071, China; 2018205528@qdu.edu.cn (H.W.); liushuo@qdu.edu.cn (S.L.)

**Keywords:** endothelial function, carbon dot-based nanosystem, mitochondrial biogenesis, intercellular mitochondrial transfer, PGC-1α/Cx43 signaling

## Abstract

Ischemic diseases are characterized by the functional collapse of endothelial cells (ECs) triggered by insufficient tissue perfusion. Given that mitochondria serve as the metabolic hub of ECs, their homeostatic imbalance, which is manifested by adenosine triphosphate (ATP) depletion, reactive oxygen species (ROS) bursts, and mitochondrial permeability transition pore opening, serves as the initiating factor driving impaired angiogenesis and tissue necrosis. In this study, we engineered an integrated nanosystem (Tan-CDs@AS-IV) by transforming Tanshinone into antioxidant carbon dots to encapsulate Astragaloside IV, achieving multi-level synergistic regulation of mitochondrial function. Our results demonstrate that Tan-CDs@AS-IV possesses superior structural stability and cellular internalization capabilities, significantly enhancing the migration and tubulogenesis of ECs under ischemic stress. Mechanistically, Tan-CDs@AS-IV effectively scavenges mitochondrial ROS and restores membrane potential and ATP production. Crucially, the nanosystem orchestrates mitochondrial biogenesis via peroxisome proliferator-activated receptor γ coactivator 1-α (PGC-1α) upregulation while simultaneously facilitating intercellular mitochondrial transfer through Connexin 43 (Cx43)-mediated gap junctions. This synergistic “endogenous amplification and intercellular replenishment” model establishes a robust mitochondrial quality control relay. By reconstructing cellular energy homeostasis, this study provides a novel nanoengineering strategy for the targeted therapy of ischemic diseases.

## 1. Introduction

Ischemic diseases represent a class of pathological conditions characterized by impaired cellular energy metabolism and functional damage resulting from insufficient tissue perfusion, with the functional collapse of vascular endothelial cells (ECs) serving as the common central feature [1]. Endothelial cells are not merely structural components of the vascular barrier but also function as critical executive units that regulate angiogenesis, blood flow homeostasis, and tissue perfusion [2]. Under the inflammatory stimulation of ischemic lesions, prolonged exposure to hypoxia, oxidative stress, and inflammatory cytokines triggers metabolism collapse, loss of proliferative and migratory capacities, and increased apoptosis in ECs, ultimately leading to the disintegration of vascular networks and the failure of collateral compensation [3,4]. Consequently, endothelial dysfunction is recognized as a pivotal factor driving ischemic tissue damage and the subsequent failure of reparative processes.

As the energetic hub of endothelial cells, persistent mitochondrial damage leads to reduced ATP production, excessive accumulation of reactive oxygen species (ROS), and the collapse of membrane potential; it also triggers cell death signals by inducing the opening of the mitochondrial permeability transition pore (mPTP), eventually hindering revascularization and accelerating tissue necrosis [5,6,7,8]. Mitochondrial homeostasis relies on precise and coordinated regulatory mechanisms. On one hand, mitochondrial biogenesis is the primary pathway for maintaining the population of functional mitochondria, a process governed by PGC-1α which promotes the formation of new mitochondria via the regulation of downstream pathways such as NRF1 and TFAM. On the other hand, mitochondrial quality control mechanisms, such as antioxidant defenses, determine the functional integrity of existing mitochondria. Studies have demonstrated that enhancing PGC-1α expression significantly bolsters mitochondrial function and reduces oxidative stress levels, thereby improving cell viability [9,10,11]. Furthermore, it enhances the metabolic adaptability of ECs, with its expression levels being closely correlated with tissue repair capacity [5].

In addition to endogenous biogenesis, intercellular mitochondrial transfer has gained increasing attention as a vital compensatory mechanism. Research indicates that under stress conditions, donor cells can transfer healthy mitochondria to damaged cells through tunneling nanotubes or gap junctions, thereby restoring energy metabolism and promoting tissue repair [12]. Among these mechanisms, Connexin 43 (Cx43) is identified as a key molecule regulating the efficiency of mitochondrial transfer, and its expression levels directly influence both transfer efficiency and the recovery of cellular function. Furthermore, Cx43 is involved not only in intercellular communication but also in the direct regulation of mitochondrial ATP production and oxidative stress responses, further reinforcing its role in ischemic repair [13,14].

Natural bioactive molecules possess significant advantages in modulating mitochondrial function. Astragaloside IV (AS-IV) has been shown to reduce ROS levels, restore mitochondrial membrane potential, and ameliorate oxidative-stress-related damage, with its effects being closely linked to mitochondrial biogenesis. In various injury models, AS-IV promotes mitochondrial biogenesis and improves energy metabolic status by modulating the SIRT1/PGC-1α axis, thereby enhancing cell survival [15]. These effects have been validated in ischemic models across various tissues, including the endothelium and myocardium [16]. Complementarily, Tanshinone (Tan) exhibits a more direct role in regulating mitochondrial oxidative stress: Tan can influence mitochondrial electron transport, reduce ROS generation, and inhibit the abnormal opening of the mPTP, thus maintaining mitochondrial structural stability [17]. However, its hydrophobicity and poor bioavailability limit its sustained efficacy within the lesion area.

Based on the aforementioned complementary mechanisms, increasing evidence suggests that single therapeutic strategies often struggle to simultaneously achieve sustained mitochondrial quality enhancement and rapid suppression of oxidative stress, due to the multi-factorial nature of mitochondrial dysfunction and ischemic injury [8,18,19]. After hydrothermal carbonization, the resulting Tan-derived carbon dots (Tan-CDs) exhibited favorable aqueous dispersibility and intracellular stability, facilitating intracellular redox regulation. In addition, the carbonized nanostructures themselves possess intrinsic antioxidant activity, which is commonly associated with their conjugated carbon domains and abundant surface functional groups [20,21,22]. The subsequent integration of AS-IV enhances mitochondrial biogenesis at the source, establishing a continuous regulatory chain of “ROS reduction—mitochondrial renewal—energy restoration”.

**Scheme 1:**
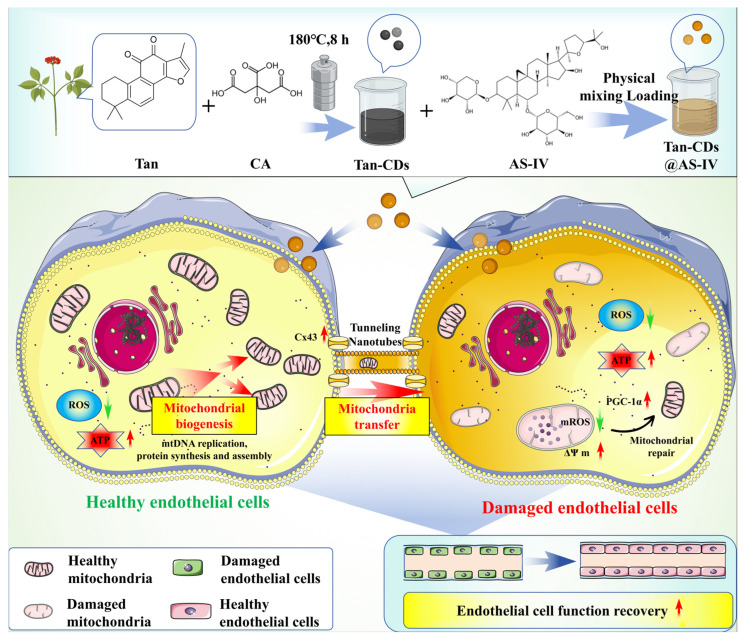
Schematic illustration of the preparation of Tan-CDs@AS-IV by physical mixing loading and its proposed mechanism in endothelial repair under ischemic conditions. Tan-CDs were synthesized through a hydrothermal reaction using Tanshinone and citric acid, followed by physical mixing with AS-IV to obtain the Tan-CDs@AS-IV nanocomposite. In the biological model, the left panel (light-colored cell) represents healthy endothelial cells with intact mitochondrial function, whereas the right panel (dark-colored cell) represents damaged endothelial cells characterized by excessive ROS accumulation, mitochondrial dysfunction, and reduced ATP production. Tan-CDs@AS-IV exerts a synergistic effect: (i) Tan-CDs directly scavenges ROS, restoring mitochondrial membrane potential and ATP production (The green arrows indicate a reducing or inhibitory effect, while the red arrows represent an increasing or promoting effect); (ii) AS-IV promotes mitochondrial biogenesis via the PGC-α pathway, leading to the generation of healthy mitochondria; (iii) Tan-CDs@AS-IV upregulates Cx43 expression and facilitates Cx43-mediated mitochondrial transfer through tunneling nanotubes, delivering functional mitochondria from donor to recipient cells. Through simultaneous regulation of mitochondrial quality, quantity and intercellular distribution, Tan-CDs@AS-IV ultimately restores endothelial energy metabolism and function.

In summary, this study innovatively designed a Tan-CDs@AS-IV nanocomposite system (Scheme 1). On one hand, this system utilizes Tan-CDs as the core functional unit, which not only improves the dispersibility and cellular uptake of Tan but also directly alleviates mitochondrial oxidative stress and restores energy metabolism through the electron transfer and antioxidant properties intrinsic to the carbon dots. On the other hand, AS-IV conjugated to the surface of the carbon dots enhances mitochondrial biogenesis via the PGC-1α pathway, while simultaneously upregulating Cx43 expression to facilitate mitochondrial transfer to damaged cells. By coordinately regulating the quality, quantity, and intercellular distribution of mitochondria, this system achieves optimal outcomes in energy metabolism recovery and cellular function improvement, providing a novel cellular-level repair strategy for the treatment of ischemic diseases.

## 2. Results and Discussion

### 2.1. Synthesis and Characterization of Tan-CDs@AS-IV

**Figure 1:**
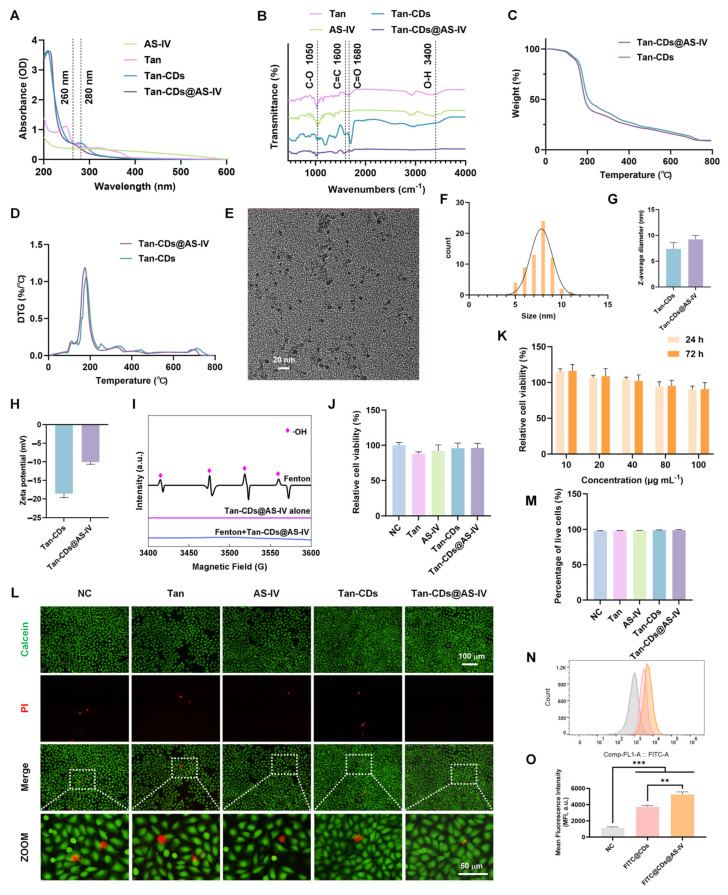
Physicochemical properties and biological activity of Tan-CDs@AS-IV. (**A**) Ultraviolet–visible absorption spectra of Tan, AS-IV, Tan-CDs and Tan-CDs@AS-IV. (**B**) FTIR spectra of Tan, AS-IV, Tan-CDs and Tan-CDs@AS-IV. (**C**) Thermogravimetric analysis (TGA) curves of Tan-CDs and Tan-CDs@AS-IV. (**D**) Differential thermogravimetric (DTG) analysis of Tan-CDs and Tan-CDs@AS-IV. (**E**) Transmission electron microscopy (TEM) image showing the morphology of Tan-CDs@AS-IV. (**F**) Particle size distribution of Tan-CDs@AS-IV statistically analyzed from TEM images (n > 100). (**G**) Dynamic light scattering (DLS) analysis showing the hydrodynamic diameter distribution of Tan-CDs@AS-IV. (**H**) Zeta potential analysis of Tan-CDs and Tan-CDs@AS-IV. (**I**) Electron paramagnetic resonance (EPR) spectra for hydroxyl radical (·OH) detection using DMPO as a spin-trapping agent in the Fenton reaction system, evaluating the ·OH scavenging capability of Tan-CDs@AS-IV and Tan-CDs@AS-IV alone. (**J**) The cytotoxicity of Tan, AS-IV, Tan-CDs and Tan-CDs@AS-IV. (**K**) Tan-CDs@AS-IV concentration gradient cytotoxicity test. (**L**) Live/dead staining of endothelial cells. (The green ones represent living cells, while the red ones represent dead cells. The dotted line box shows a magnified view of a local area.) (**M**) Quantitative analysis of live cell percentage. (**N**) Flow cytometry histograms showing cellular uptake of FITC-labeled nanodrugs. (Orange represents FITC@CDs@AS-IV, red represents FITC@CDs, and gray represents the control group without any drug addition.) (**O**) Mean fluorescence intensity quantified by flow cytometry. Data represent mean ± SD. ** *p* < 0.01, *** *p* < 0.001.

To restore endothelial mitochondrial function, Tan-derived carbon quantum dots loaded with Astragaloside IV (Tan-CDs@AS-IV) were synthesized via a green hydrothermal strategy and systematically characterized. As shown in Figure 1A, pristine Tan exhibited a characteristic absorption peak at 250 nm with tailing near 320 nm, attributed to the π–π* and n–π* transitions of its quinone-conjugated structure. After hydrothermal treatment, this characteristic peak disappeared and was replaced by a broad absorption band centered around 280 nm, indicating the formation of sp2-hybridized carbon domains and heterogeneous surface states during carbonization [23]. These spectral changes suggest that the native molecular structure of Tan was not preserved, but instead underwent thermal degradation and structural reconstruction to form Tan-derived carbon dots. Following AS-IV loading, the broad absorption band of Tan-CDs shifted from 280 nm to approximately 260 nm with reduced absorption intensity. This blue shift is likely associated with interactions between AS-IV and the surface functional groups of Tan-CDs, leading to alterations in the local electronic environment and surface-state transitions of the carbon dots. The absence of recovery of the original Tan absorption profile further confirms that the carbonized core structure remained stable after AS-IV incorporation.

FTIR spectroscopy was performed to further verify the successful loading of AS-IV onto Tan-CDs (Figure 1B). Compared with Tan-CDs, the FTIR spectrum of Tan-CDs@AS-IV exhibited obvious changes in several characteristic absorption regions. In particular, the absorption bands around 1680 cm^−1^ and 1600 cm^−1^, corresponding to C=O stretching vibration and aromatic C=C vibration in Tan-CDs, became significantly weakened after AS-IV loading, indicating that the surface chemical environment of Tan-CDs changed after incorporation of AS-IV. In addition, slight changes were also observed in the C–O-related absorption region around 1050 cm^−1^. These spectral differences suggest that AS-IV was successfully loaded onto the surface of Tan-CDs. Notably, no distinct new absorption peaks corresponding to newly formed covalent bonds were observed, indicating that the loading process was more likely based on non-covalent interactions rather than covalent chemical conjugation.

Thermogravimetric analysis (TGA) was further performed to evaluate the successful loading of AS-IV onto Tan-CDs. TGA revealed distinct (Figure 1C) thermal decomposition behaviors between Tan-CDs and Tan-CDs@AS-IV. Compared with Tan-CDs, Tan-CDs@AS-IV exhibited an increased weight-loss rate in the 150–250 °C range and slightly higher residual mass at elevated temperatures, indicating successful incorporation of AS-IV into the nanosystem. Differential thermogravimetric (DTG) analysis (Figure 1D) further demonstrated enhanced decomposition peaks in Tan-CDs@AS-IV, suggesting the contribution of additional organic components derived from AS-IV. Based on the differential weight loss within the main decomposition region, the loading content of AS-IV was estimated to be approximately 6 wt%. These results, together with FTIR and UV–Vis analyses, support that AS-IV was effectively loaded onto Tan-CDs mainly through non-covalent interactions.

Transmission electron microscopy (TEM, JEOL JEM-F200, Tokyo, Japan) was employed to characterize the morphology and surface state of the composite nanoparticles. TEM results reveal that Tan-CDs@AS-IV consists of monodispersed, spherical-like nanoparticles without obvious agglomeration, demonstrating that the system maintains excellent colloidal stability following the introduction of AS-IV (Figure 1E). The size distribution statistically obtained (n > 100) further confirmed the nanoscale consistency of the system (Figure 1F). Previous studies have shown that carbon dots are typically nanoparticles with sizes less than 10 nm and possess good dispersibility, which is closely related to the abundance of functional groups on their surface [24]. Dynamic light scattering (DLS) analysis further confirmed an average hydrodynamic diameter of approximately 8 nm for Tan-CDs@AS-IV (Figure 1G), consistent with TEM observations. In addition, the zeta potential values of Tan-CDs and Tan-CDs@AS-IV were measured to be approximately −18 mV and −10 mV (Figure 1H), respectively, indicating successful surface modification with AS-IV and altered surface charge distribution.

To further evaluate the intrinsic ROS-scavenging capability of the nanosystem, electron paramagnetic resonance (EPR) analysis was performed using DMPO as a spin-trapping agent for hydroxyl radicals (·OH). As shown in Figure 1I, the radical-generating system exhibited a characteristic DMPO/·OH quartet signal, whereas the signal intensity was markedly attenuated after treatment with Tan-CDs@AS-IV, indicating effective ·OH scavenging activity. In addition, EPR analysis of Tan-CDs@AS-IV alone in the absence of an exogenous ROS-generating system did not reveal obvious radical-related signals, suggesting that the nanosystem itself was not a major source of stable radicals under the experimental conditions.

Taken together, the spectroscopic and morphological characterizations confirm the successful construction of a structurally stable Tan-CDs@AS-IV nanosystem in this study. This engineered nanosystem integrates the physicochemical characteristics of Tan-CDs with the mitochondrial regulatory activity of AS-IV, thereby providing a robust foundation for intracellular ROS regulation and mitochondrial functional recovery.

### 2.2. Cytocompatibility and Cellular Uptake of Tan-CDs@AS-IV

Prior to investigating mitochondrial functional repair, evaluating the biosafety and cellular internalization efficiency of the nanosystem is paramount. In this study, HUVECs were utilized as model cells to systematically evaluate the biological effects of free drugs and the nanosystem via CCK-8 assay, live/dead cell staining, and flow cytometry.

Results indicated that at a concentration of 100 μg/mL, none of the treatment groups induced significant cytotoxicity; however, distinct differences were observed among the various delivery formats (Figure 1J). In the free Tan group, cell viability decreased slightly to approximately 85–90% at this concentration, whereas the survival rate in the Tan-CDs group improved to approximately 90–95%. Meanwhile, the free AS-IV group maintained a high survival level (88–92%), while the Tan-CDs@AS-IV composite system exhibited more stable cellular tolerance, with survival rates consistently remaining above 95% and no trend toward toxicity accumulation observed even at high doses. Meanwhile, the cytocompatibility of Tan-CDs@AS-IV was further evaluated over a concentration range of 10–100 μg/mL at both 24 h and 72 h. The results showed that cell viability remained above 90% across all tested concentrations and incubation periods, even at the highest concentration of 100 μg/mL (Figure 1K). Moreover, no obvious time-dependent cytotoxicity or toxicity accumulation was observed after prolonged incubation, indicating favorable biosafety and stability of the nanosystem in endothelial cells. Consistently, live/dead staining further confirmed that Tan-CDs@AS-IV did not induce significant cell death under the tested conditions (Figure 1L,M).

The therapeutic efficacy of nanodelivery systems relies heavily on their cellular internalization. This study compared the uptake behaviors of FITC-labeled Tan-CDs and Tan-CDs@AS-IV in HUVECs using flow cytometry. The results showed that the mean fluorescence intensity of the Tan-CDs@AS-IV treatment group was approximately 1.4 times that of the Tan-CDs group, indicating higher efficiency in cellular internalization for the composite system (Figure 1N,O). This difference may be associated with changes in surface physicochemical properties after AS-IV incorporation. However, the detailed mechanisms underlying the enhanced cellular uptake require further investigation.

These experiments demonstrate that the Tan-CDs@AS-IV system significantly improves cellular uptake efficiency while maintaining excellent cytocompatibility. This synergistic feature of “low toxicity and high uptake” provides a necessary prerequisite for its effective performance in subsequent mitochondrial functional regulation.

### 2.3. Tan-CDs@AS-IV Enhances the Antioxidant and Angiogenic Capacities of Endothelial Cells

The functional state of endothelial cells within the ischemic microenvironment directly dictates the success or failure of tissue repair. This study first evaluated the regulatory effects of various treatments on intracellular oxidative stress levels using a lipopolysaccharide (LPS)-induced inflammatory injury model.

**Figure 2:**
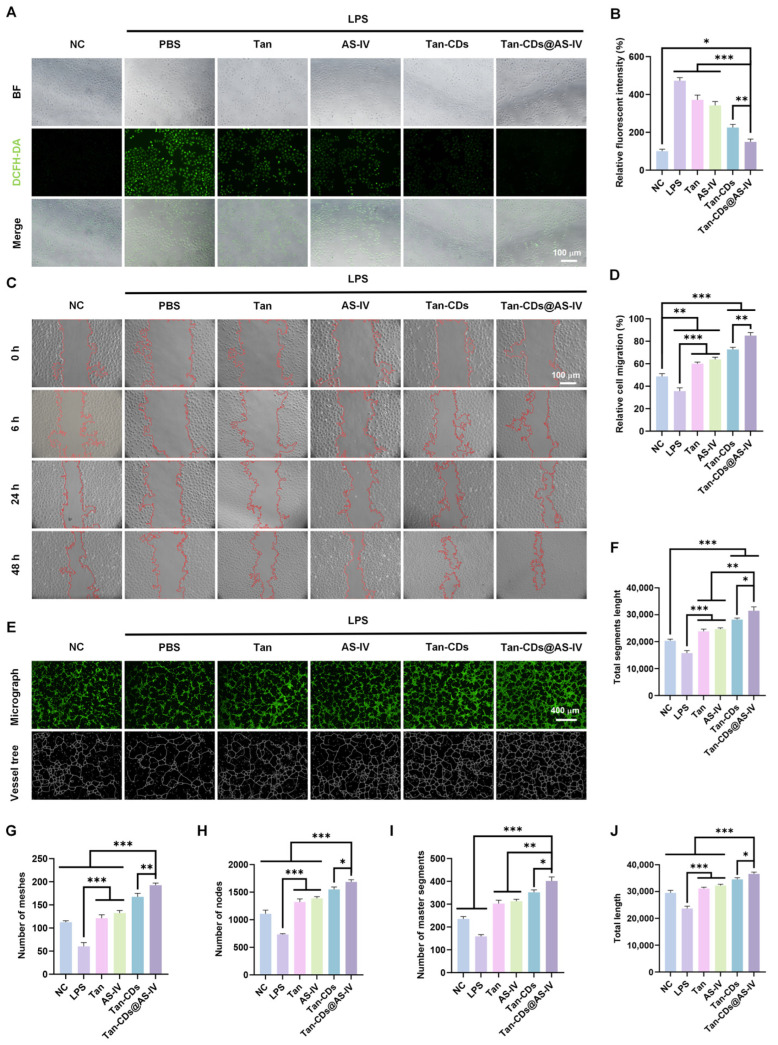
Tan-CDs@AS-IV enhances the function of ECs. (**A**) Representative regions and morphological changes of intracellular ROS in ECs. (**B**) Quantitative analysis of intracellular ROS levels in ECs detected using the 2′,7′-dichlorodihydrofluorescein diacetate (DCFH-DA) probe. (**C**) Representative images of ECs migration. (**D**) Quantitative analysis of ECs migration. (**E**) Endothelial tube formation images. (**F**–**J**) Quantitative analysis of total segments length, number of meshes, number of nodes, number of master segments and total length. Data represent mean ± SD. * *p* < 0.05, ** *p* < 0.01, *** *p* < 0.001.

The excessive accumulation of ROS is a core factor contributing to endothelial dysfunction. DCFH-DA staining results (Figure 2A,B) revealed that LPS stimulation significantly elevated intracellular ROS levels. While the free Tan and AS-IV groups exhibited relatively limited ROS scavenging effects, the Tan-CDs group showed a significant inhibitory effect, suppressing LPS-induced ROS upregulation by approximately 66.7%. This effect may be associated with the intrinsic antioxidant activity and ROS scavenging capability of the carbonized nanostructures themselves [25,26]. Notably, the Tan-CDs@AS-IV composite system demonstrated the most superior antioxidant capacity, inhibiting LPS-induced ROS upregulation by approximately 87.1% and restoring the overall fluorescence intensity to levels near those of the negative control. This synergistic effect confirms the complementary roles of AS-IV and Tan-CDs in the regulation of redox homeostasis.

The process of angiogenesis depends on the migration and structural remodeling of endothelial cells, which were assessed in this study via scratch migration and tube formation assays, respectively. Scratch assay results (Figure 2C,D) showed that, comparing the scratch area at 48 h to the initial state, the migration rate of the LPS-induced group was approximately 48%; this increased to ~60% in the free Tan group, ~63% in the free AS-IV group, and further to ~72% in the Tan-CDs group. Strikingly, the migration capacity of the Tan-CDs@AS-IV group was significantly enhanced, achieving a scratch closure rate of approximately 85%. These findings demonstrate that the composite system not only improves cell viability but also significantly bolsters cellular migratory capacity. Tube formation assays further revealed the stimulatory effect of the composite system on vascular network reconstruction. Results (Figure 2E–J) indicated that, compared to the LPS-induced group, endothelial cells treated with the Tan-CDs@AS-IV composite system formed more dense and continuous tube-like networks. Specifically, the treatment led to an approximately 3-fold increase in the number of meshes, a 2.2-fold increase in junction points, and an over 1.5-fold increase in total segment length, indicating significantly enhanced angiogenic potential.

Taken together, these results indicate that Tan-CDs@AS-IV not only serves as an efficient ROS scavenger to alleviate oxidative stress but also significantly enhances migration and angiogenic potential under stress by remodeling the biological behavior of endothelial cells. This functional improvement likely stems from the intimate coupling between mitochondrial homeostatic restoration and angiogenic signaling pathways. On one hand, the improvement in mitochondrial homeostasis mediated by Tan-CDs@AS-IV enhances ATP production efficiency, providing sustained energy support for cell migration and cytoskeletal rearrangement. On the other hand, the reduction in ROS load helps maintain the activity of angiogenic signals such as vascular endothelial growth factor (VEGF), preventing their inactivation in an oxidative environment. Recent research has noted that mitochondria are not only energy factories but also critical signaling platforms regulating endothelial cell fate; mitochondrial damage affects not only energy supply but also inhibits angiogenesis by modulating cellular signaling networks [27,28].

### 2.4. Tan-CDs@AS-IV Mediated Mitochondrial Structural Repair and Energy Metabolism Reconstruction

Ischemia–reperfusion environments can induce a decline in the electron transfer efficiency of the mitochondrial respiratory chain, leading to increased electron leakage and the generation of excessive ROS, which further triggers lipid peroxidation and mitochondrial membrane structural destruction—a pivotal driver of endothelial functional impairment. Consequently, reducing mitochondrial ROS (mROS) levels may exert a beneficial promoting effect on the restoration of endothelial function and angiogenesis [29,30]. To systematically evaluate the intervention effects of various treatment systems on mitochondrial oxidative damage, this study performed functional assessments using mitochondrial-specific ROS detection (MitoSOX) and membrane potential analysis (JC-1); simultaneously, the mitochondrial outer membrane marker protein TOM20 and ATP production levels were introduced to comprehensively analyze the degree of mitochondrial repair across both structural and metabolic dimensions.

**Figure 3:**
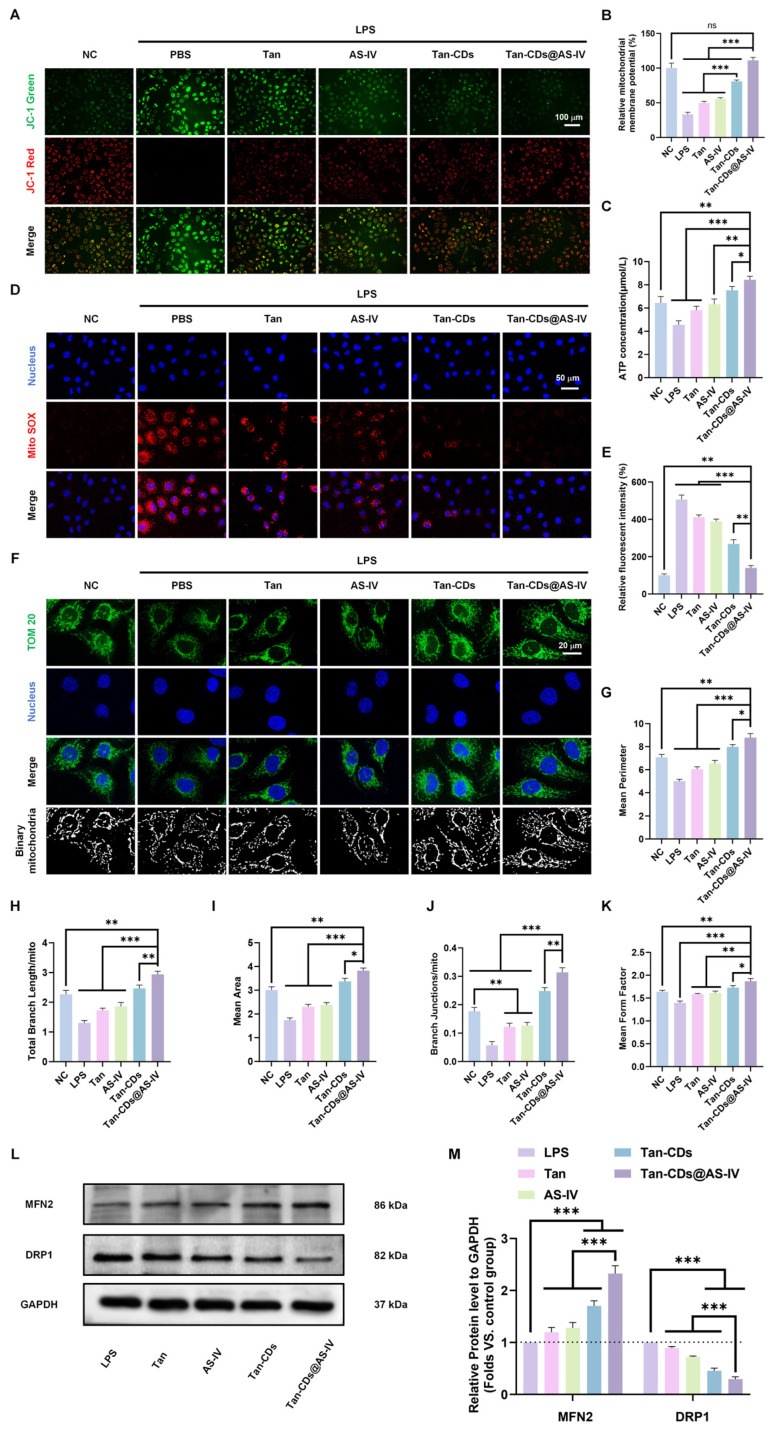
Tan-CDs@AS-IV Restores Mitochondrial Function and Structural Integrity in Endothelial Cells. (**A**) The mitochondrial membrane potential (MMP) detected by JC-1 dye shows that red fluorescence represents intact MMP, while green fluorescence indicates depolarized mitochondria. (**B**) The ratio of red fluorescence intensity to green fluorescence intensity. (**C**) Quantitative analysis of ATP levels within different groups of cells. (**D**) The level of mROS detected by the MitoSOX Red fluorescent probe (**E**) Quantitative analysis of mROS levels in different treatment groups. (**F**) The immunofluorescence staining of TOM20 in different cell groups revealed changes in the morphology of the mitochondrial network. (**G**–**K**) Quantitative analysis of mitochondrial morphology based on TOM20-stained images, including: (**G**) average mitochondrial perimeter, (**H**) average mitochondrial length, (**I**) average mitochondrial area, (**J**) average number of branch junctions per mitochondrion, and (**K**) mitochondrial shape factor. Morphological parameters were extracted using ImageJ-based mitochondrial network analysis. (**L**) Western blot bands showing protein expression of MFN2, DRP1 and glyceraldehyde-3-phosphate dehydrogenase (GAPDH). (**M**) Quantitative analysis of the above protein expression levels (The dotted lines represent the ratios of the expression levels of each pathway protein in the LPS model group to GAPDH, which is set as 1. The other treatment groups are then compared with this value). Data represent mean ± SD. * *p* < 0.05, ** *p* < 0.01, *** *p* < 0.001. ns = not significant.

Mitochondrial transmembrane potential (ΔΨm) serves as the physical foundation for oxidative phosphorylation-driven ATP synthesis. JC-1 assay results (Figure 3A,B) revealed a significant decrease in the red-to-green fluorescence ratio in the LPS-induced group, indicating mitochondrial depolarization; although the free drug groups partially restored the membrane potential, it only recovered to 49–55% of normal levels. In contrast, Tan-CDs@AS-IV treatment significantly enhanced red aggregate fluorescence, with the red-to-green ratio restored to near-baseline levels (~110% of the control), indicating that the mitochondrial transmembrane potential gradient was essentially restored. The stability of the membrane potential is a prerequisite for the normal operation of oxidative phosphorylation, and its restoration signifies that the electron transport chain and ATP synthase complex have returned to a functional state. Further ATP quantitative analysis (Figure 3C) revealed that intracellular ATP levels in the composite system treatment group increased approximately 1.8-fold compared to the model group, which was significantly higher than the levels observed in the single-drug groups. These lines of evidence suggest that mitochondrial reconstruction is manifested not only in the maintenance of membrane potential homeostasis but also in the transformation into energy output, providing kinetic support for cellular physiological functions [27].

MitoSOX assay results (Figure 3D,E) demonstrated that the Tan-CDs@AS-IV system significantly inhibited mitochondrial superoxide anion generation, suppressing LPS-induced mROS upregulation by approximately 90.4%, a result markedly superior to those of the single-drug treatment groups. This indicates that the composite system is more efficient in modulating mitochondrial oxidative stress; acting as a functional nanounit, it preserves the bioactivity of Tan while endowing the system with excellent electron transfer and radical scavenging capabilities, thereby achieving efficient response and clearance of mROS within the intracellular mitochondrial microenvironment. Meanwhile, the grafting of AS-IV further enhances the regulatory effect of the system on mitochondrial function, potentially reducing abnormal ROS production at the source by activating PGC-1α-mediated mitochondrial biogenesis and improving CX43-related intercellular mitochondrial communication.

The distribution characteristics of the translocase of the outer mitochondrial membrane 20 (TOM20) sensitively reflect the morphodynamic state of mitochondria. Immunofluorescence results (Figure 3F–K) showed that under LPS stimulation, mitochondria exhibited a fragmented distribution with a disintegrated network structure, and the average mitochondrial area decreased by approximately 44% compared to the control group. In contrast, for endothelial cells treated with Tan-CDs@AS-IV, the average mitochondrial area rose to 119% of the control group level, and both the average mitochondrial length and form factor were significantly improved. Through quantitative analysis of the images using ImageJ, the key morphological parameters were obtained, including the average mitochondrial perimeter (Figure 3G), the average mitochondrial length (Figure 3H), the average mitochondrial area (Figure 3I), the average number of branch connection points per mitochondrion (Figure 3J), and the mitochondrial form factor (Figure 3K). These results collectively indicate that Tan-CDs@AS-IV can effectively restore the integrity and structural connectivity of the mitochondrial network under inflammatory stress. Such structural remodeling is crucial for maintaining angiogenic signaling, as an intact mitochondrial network has been proven to be a prerequisite for endothelial cells to maintain migration and tube formation capabilities under stress [31].

To further investigate mitochondrial structural regulation, the expression levels of the mitochondrial-dynamics-related proteins DRP1 and MFN2 were examined (Figure 3L,M). Compared with the LPS group, Tan-CDs@AS-IV treatment markedly altered the expression patterns of these proteins, characterized by a reduction in DRP1 expression and an increase in MFN2 expression. These results suggest that Tan-CDs@AS-IV may contribute to the restoration of mitochondrial fusion–fission balance under inflammatory stress conditions, further supporting the observed improvement in mitochondrial network integrity.

Integrating the sequential evidence chain of “TOM20 structural recovery, membrane potential reconstruction, and enhanced ATP production”, these experiments demonstrate that Tan-CDs@AS-IV functions not only at the level of oxidative stress but also collectively suggest that Tan-CDs@AS-IV contributes to the restoration of mitochondrial structural integrity and functional activity under inflammatory stress conditions.

### 2.5. Tan-CDs@AS-IV Promotes Intercellular Mitochondrial Transfer via the PGC-1α/Cx43 Axis

Intercellular mitochondrial transfer has emerged as a critical biological compensatory mechanism, whereby damaged cells acquire functional mitochondria from neighboring donor cells to rapidly restore oxidative phosphorylation capacity and maintain energy homeostasis [28]. In this study, fluorescence-labeled mitochondrial tracing techniques, including Mito-Tracker staining and co-culture systems, were employed to systematically evaluate the regulatory effects of different treatment groups on the flux of intercellular mitochondrial transfer.

**Figure 4:**
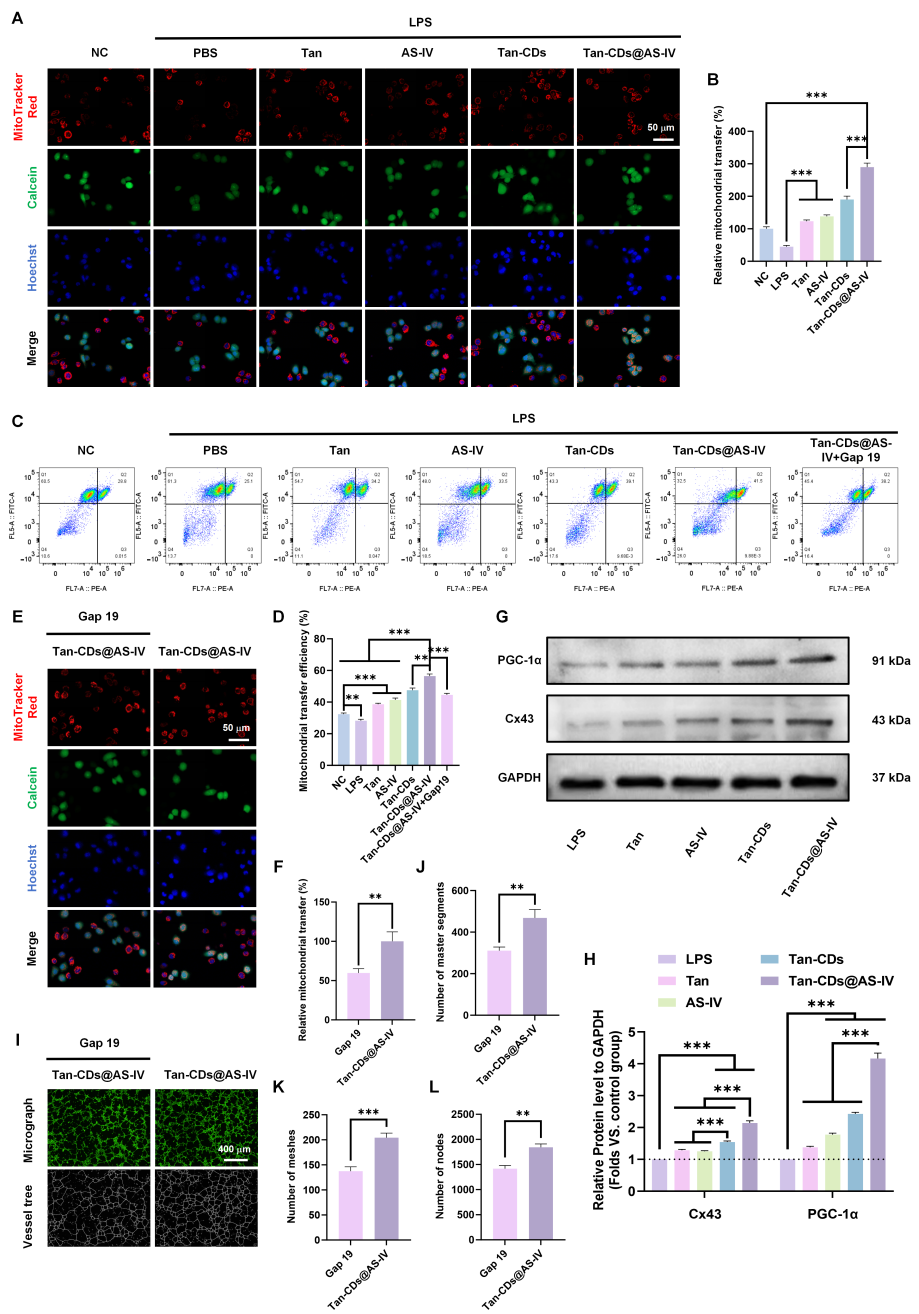
Tan-CDs@AS-IV enhances intercellular mitochondrial transfer via the PGC-1α/Cx43 axis. (**A**) Observation of the transfer of mitochondria from healthy endothelial cells to damaged endothelial cells after treatment with different drugs was conducted using confocal microscopy. (**B**) The fluorescence intensity of the mitochondria involved in the reaction transfer was quantitatively analyzed. (**C**) Flow cytometry scatter plots showing mitochondrial transfer efficiency among different treatment groups (The pseudocolor scale indicates cell density, with red representing a higher density of events and blue representing a lower density of events). (**D**) Quantitative analysis of double-positive cell populations involved in mitochondrial transfer. (**E**) Confocal fluorescence images showing mitochondrial transfer in the Tan-CDs@AS-IV group and the Gap19 + Tan-CDs@AS-IV group. (**F**) Quantitative analysis of mitochondrial transfer-associated fluorescence intensity in the Tan-CDs@AS-IV group and the Gap19 + Tan-CDs@AS-IV group. (**G**) Western blot bands showing protein expression of PGC-1α, Cx43 and glyceraldehyde-3-phosphate dehydrogenase (GAPDH). (**H**) Quantitative analysis of the above protein expression levels (The dotted lines represent the ratios of the expression levels of each pathway protein in the LPS model group to GAPDH, which is set as 1. The other treatment groups are then compared with this value). (**I**) Representative images of endothelial tube formation in the Tan-CDs@AS-IV group and the Gap19 + Tan-CDs@AS-IV group. (**J**–**L**) Quantitative analysis of number of master segments, number of meshes and number of nodes. Data represent mean ± SD. ** *p* < 0.01, *** *p* < 0.001.

Co-culture experiments between donor and recipient cells (both subjected to LPS-induced oxidative damage) were conducted to observe mitochondrial transfer. Confocal imaging (Figure 4A,B) revealed that mitochondrial transfer from donor cells in the LPS-treated group without drug intervention was significantly restricted, with almost no exchange of fluorescent signals observed, demonstrating that the injury microenvironment severely inhibits intercellular organelle communication. In contrast, normal donor cells without LPS treatment were capable of transferring mitochondria to damaged recipient cells. Following treatment with Tan and AS-IV, mitochondrial transfer efficiency increased slightly; compared to the normal donor cell group without LPS, fluorescent signal intensity rose by approximately 23–38%, suggesting that while an improved antioxidant environment helps maintain mitochondrial structural stability, it is insufficient to effectively activate intercellular transport pathways. The Tan-CDs group exhibited higher transfer efficiency, with fluorescence intensity increasing by approximately 89.6% compared to the normal donor cell group without LPS. The Tan-CDs@AS-IV composite system showed the most significant promotion effect, with mitochondrial transfer-related fluorescence intensity increasing by approximately 189.3% relative to the normal donor group without LPS.

Quantitative flow cytometric analysis was further performed across seven experimental groups, including CN, LPS, Tan, AS-IV, Tan-CDs, Tan-CDs@AS-IV, and Gap19 + Tan-CDs@AS-IV. The results demonstrated (Figure 4C,D) that LPS stimulation significantly reduced mitochondrial transfer efficiency compared with the CN group. Free Tan and AS-IV groups showed limited improvement, whereas Tan-CDs enhanced mitochondrial transfer to a greater extent. Notably, Tan-CDs@AS-IV exhibited the highest level of mitochondrial transfer among all treatment groups, as reflected by a marked increase in double-positive cell populations. Importantly, inhibition of Cx43 using Gap19 significantly reversed the enhanced mitochondrial transfer induced by Tan-CDs@AS-IV (Figure 4E,F), indicating that this process is at least partially dependent on Cx43-mediated intercellular communication.

Efficient mitochondrial transfer depends on an adequate source of mitochondria (biogenesis) and unobstructed transport pathways (gap junctions). PGC-1α is recognized as the core transcriptional coactivator regulating mitochondrial biogenesis, while Cx43 serves as a critical channel in intercellular communication and mitochondrial transfer processes [11,32]. To this end, the expression levels of the key regulatory proteins PGC-1α and Cx43 were detected via Western blot.

The results showed (Figure 4G,H) that PGC-1α expression was weak in the LPS group; the free drug groups induced slight upregulation (approx. 1.3–1.8 fold), the Tan-CDs group increased it to ~2.4 fold, and Tan-CDs@AS-IV treatment significantly elevated PGC-1α expression to ~4.1 fold, demonstrating a markedly enhanced capacity for mitochondrial biogenesis. The expression trend of the Cx43 protein was largely consistent with that of PGC-1α. Following Tan-CDs@AS-IV treatment, Cx43 expression increased by approximately 2.3-fold, suggesting a significant enhancement in intercellular gap junctions. As a classic gap junction protein, Cx43 not only mediates the intercellular transfer of small molecules and ions but has also been confirmed to participate in mitochondrial transfer, providing functional mitochondrial replenishment to damaged cells. Elevated Cx43 expression levels can enhance intercellular communication efficiency and promote mitochondrial transport via tunneling nanotubes (TNTs) or extracellular vesicles, thereby improving the energy metabolic state of damaged tissues [33].

To further evaluate the functional involvement of Cx43-mediated mitochondrial transfer, a tube formation assay was additionally performed in the presence of the Cx43 inhibitor Gap19. As shown in Figure 4I, Tan-CDs@AS-IV treatment markedly promoted endothelial tube formation, whereas this pro-angiogenic effect was significantly attenuated following Gap19 treatment. Quantitative analysis demonstrated (Figure 4J–L) obvious reductions in mesh number, junction points, and total segment length after Cx43 inhibition. These findings were generally consistent with the changes observed in mitochondrial transfer efficiency, further suggesting that Cx43-associated intercellular communication may contribute to the vascular protective effects mediated by Tan-CDs@AS-IV.

Integrating these findings, it can be inferred that the Tan-CDs@AS-IV composite system modulates mitochondrial function through a multi-level synergistic mechanism rather than a single pathway. On one hand, it activates the PGC-1α pathway to promote mitochondrial biogenesis, increasing intracellular mitochondrial population and functional reserves. On the other hand, it upregulates Cx43 to enhance intercellular connectivity, providing the structural foundation for intercellular mitochondrial transfer. This dual regulatory mode of “endogenous generation plus exogenous supplementation” facilitates the rapid restoration of cellular energy metabolic homeostasis under conditions of ischemia and oxidative stress.

## 3. Conclusions

Aiming at the core pathological process of vascular endothelial functional collapse in ischemic diseases, this study successfully constructed an engineered nanosystem (Tan-CDs@AS-IV) with multi-dimensional mitochondrial repair capabilities. In vitro results demonstrate that Tan-CDs@AS-IV effectively restores mitochondrial homeostasis in endothelial cells, as evidenced by reduced mitochondrial ROS, improved mitochondrial network integrity, and enhanced ATP production, as well as angiogenic-related cellular functions. Mechanistically, these effects are mainly associated with the synergistic activation of PGC-1α-mediated mitochondrial biogenesis and Cx43-dependent intercellular mitochondrial transfer. While these findings provide mechanistic insight into mitochondrial remodeling in endothelial protection, it should be noted that the present study is limited to in vitro cellular models, and the complexity of in vivo ischemic microenvironment as well as additional regulatory mechanisms remains to be further investigated. Overall, this work presents a mitochondrial-targeted nanoplatform that may offer a conceptual basis for vascular repair strategies.

## 4. Experimental Section

### 4.1. Preparation of Tan-CDs

Tan-CDs were synthesized via a modified hydrothermal method to improve the aqueous solubility of Tan and establish a nanocarrier platform. Briefly, 500 mg of Tan powder was accurately weighed and transferred into a 100 mL three-neck flask. Subsequently, 80 mL of deionized water was added, followed by the slow addition of 20 mL of absolute ethanol to obtain an initial solvent ratio of water: ethanol = 4:1. The mixture was stirred at 400 rpm for 15 min until a homogeneous solution was formed. Citric acid (500 mg) was then introduced as an auxiliary carbon source, and the mixture was further stirred for 30 min to ensure sufficient interaction between Tan and citric acid. The flask was tightly sealed, and the solution was transferred into a Teflon-lined stainless steel autoclave, leaving at least one-fifth of the volume unfilled.

The autoclave was heated at 180 °C for 10 h in a drying oven. After natural cooling to room temperature, the resulting dark brown solution was collected and transferred into 50 mL centrifuge tubes. The suspension was centrifuged at 12,000 rpm for 15 min at 4 °C, and the precipitate was discarded. The supernatant was then filtered through a 0.22 μm membrane to remove unreacted materials and large particles.

The filtrate was subjected to dialysis using a 1 kDa dialysis membrane against 2 L of deionized water for 24 h, with the dialysis medium refreshed every 6 h, to remove small-molecule impurities and residual precursors. The resulting transparent brownish–yellow solution was freeze-dried (12 h pre-freezing followed by 48 h lyophilization) to obtain dry Tan-CDs powder. The final product appeared as a dark brown powder with good water solubility. A stock solution (1 mg/mL) was prepared using ultrapure water and stored at 4 °C in the dark until use.

### 4.2. Preparation of Tan-CDs@AS-IV

To construct the composite nanodrug system, AS-IV was loaded onto Tan-CDs via a non-covalent adsorption method. Briefly, 100 mg of Tan-CDs powder was dispersed in 12 mL of 0.1 M MES buffer (pH 5.5) and ultrasonicated for 10 min to obtain a homogeneous suspension. Separately, 50 mg of AS-IV was dissolved in 5 mL of DMSO and sonicated for 10 min until a clear solution was obtained. The AS-IV solution was then added dropwise into the Tan-CDs dispersion under continuous stirring at 25 °C and 300 rpm in the dark. The mixture was further stirred for 24 h to allow sufficient drug adsorption through non-covalent interactions. After incubation, the mixture was centrifuged at 10,000 rpm for 15 min to remove potential aggregates. The supernatant was collected and transferred into a 1 kDa dialysis bag and dialyzed against 2 L of deionized water for 24 h, with water replaced every 6 h to remove unbound AS-IV. Finally, the purified Tan-CDs@AS-IV nanocomplex was obtained by lyophilization. The final product was redispersed in deionized water to a concentration of 1 mg/mL, aliquoted, and stored at 4 °C in the dark for further use.

### 4.3. Preparation of FITC@Tan-CDs and FITC@Tan-CDs@AS-IV

A total of 5 mg of Tan-CDs or Tan-CDs@AS-IV was weighed and dissolved in a 2 mL mixture of carbonate buffer (pH 8.5) and dimethyl sulfoxide (1:1, *v*/*v*). To this solution, 5 mg of EDCI and 3 mg of FITC-NH2 were added, and the mixture was stirred under a nitrogen (N2) atmosphere for 72 h. Subsequently, 2 mL of an ethanol solution containing 3 wt% NaOH was added, and the reaction was stirred for an additional 4 h. The pH of the solution was then adjusted to neutral. The resulting solution was transferred to a 500 Da dialysis bag for 24 h, followed by freezing at −80 °C for 2 h, and it was finally lyophilized for 48 h to obtain FITC@Tan-CDs or FITC@Tan-CDs@AS-IV.

### 4.4. CCK-8 Assay for Cytotoxicity Evaluation

Cell viability was assessed using a CCK-8 assay (Meilun Biotechnology Co., Ltd., Dalian, China). HUVECs were digested with trypsin and seeded into 96-well plates at a density of 1 × 10^4^ cells per well, with six replicates for each group. PBS was added to peripheral wells to minimize evaporation. Cells were cultured at 37 °C in a humidified incubator with 5% CO_2_ for 24 h to allow full attachment. After incubation, the medium was replaced with fresh complete medium containing different concentrations (0, 10, 20, 40, 80, and 100 μg/mL) of the following formulations: (i) Tan, (ii) AS-IV, (iii) Tan-CDs, and (iv) Tan-CDs@AS-IV (100 μL per well). After 24 h of treatment, the medium was removed, and 100 μL of complete medium containing 5% CCK-8 reagent was added to each well. Cells were incubated for 1 h at 37 °C in the dark. The absorbance at 450 nm was measured using a microplate reader. Cell viability was calculated as follows:

Cell viability (%) = (OD_sample − OD_blank)/(OD_control − OD_blank) × 100%

### 4.5. Cellular Uptake Analysis by Flow Cytometry

To compare cellular uptake efficiency, HUVECs were seeded into 6-well plates at a density of 2 × 10^5^ cells per well and cultured for 24 h. After attachment, the medium was replaced with fresh medium containing FITC@Tan-CDs or FITC@Tan-CDs@AS-IV at a final concentration of 10 μg/mL. After 3 h of incubation, the medium was removed, and the cells were washed three times with PBS to eliminate non-internalized nanoparticles. Cells were then trypsinized, collected into 5 mL flow cytometry tubes, and resuspended in 500 μL PBS. Fluorescence intensity was measured using a BD FACSCalibur flow cytometer (BD Biosciences, San Jose, CA, USA). A total of 10,000 cells were recorded for each sample, and the mean fluorescence intensity was analyzed using FlowJo (version 10.9.0) software.

### 4.6. Intracellular ROS Detection

Intracellular ROS levels were measured using the DCFH-DA fluorescent probe (Solarbio Life Sciences, Beijing, China). HUVECs were seeded into 24-well plates at a density of 3 × 10^4^ cells per well, with three replicates per group. PBS was added to the outer wells to reduce evaporation. Cells were cultured at 37 °C with 5% CO_2_ for 24 h. To establish the inflammatory model, cells were treated with 1 μg/mL LPS for 24 h, while untreated cells served as the control group. For intervention, the LPS-treated groups received either PBS (25 μL, positive control) or drug formulations (25 μL, final concentration 10 μg/mL). After 18 h of treatment, the medium was removed, and cells were incubated with DCFH-DA working solution (final concentration 2 μM) at 37 °C in the dark for 30 min. The plate was gently shaken every 10 min to ensure uniform staining. Cells were then washed three times with PBS to remove excess probe. Fluorescence images were acquired using an inverted fluorescence microscope (Ex/Em = 488/525 nm), with five random fields captured per well. Fluorescence intensity was semi-quantitatively analyzed using ImageJ (version 1.54) software to evaluate intracellular ROS levels.

### 4.7. TOM20 Immunofluorescence Staining

HUVECs were seeded into confocal dishes at a density of 1 × 10^4^ cells per well, with three replicates per group. After 24 h of incubation to allow cell attachment, oxidative stress was induced by treatment with LPS for 4 h (control group received an equal volume of PBS). After 24 h, the medium was removed and replaced with fresh medium containing the corresponding treatments for an additional 24 h (LPS model and control groups received PBS). Cells were then washed three times with PBS and fixed with 4% paraformaldehyde at room temperature for 15 min, followed by three PBS washes. Cells were permeabilized with 0.1% Triton X-100 (Sinopharm Chemical Reagent Co., Ltd., Shanghai, China) for 10 min and washed again. Subsequently, cells were blocked with 5% BSA for 30 min at room temperature to reduce non-specific binding.

After removing the blocking solution, cells were incubated with primary antibody against TOM20 (1:200) overnight at 4 °C. The following day, samples were returned to room temperature and washed three times with PBS (5 min each). Fluorescent secondary antibody (1:500) was then applied and incubated for 1 h in the dark. After washing, nuclei were counterstained with DAPI for 5 min, and samples were mounted with antifade mounting medium. Images were acquired using a laser scanning confocal microscope, with five random fields selected per sample. Mitochondrial number, length, and branching were quantified using ImageJ software.

### 4.8. JC-1 Assay for Mitochondrial Membrane Potential

Mitochondrial membrane potential (ΔΨm) was assessed using the JC-1 fluorescent probe. HUVECs were seeded into 24-well plates at a density of 2 × 10^4^ cells per well and cultured for 24 h. Oxidative stress was induced using LPS (control group received PBS). After 18 h, cells were treated with four formulations at a final concentration of 10 μg/mL (LPS model and control groups received PBS). Following 24 h of treatment, the medium was removed and replaced with 500 μL of JC-1 staining solution per well. Cells were incubated at 37 °C in the dark for 20 min, followed by nuclear staining with Hoechst. After staining, cells were washed twice with JC-1 buffer. Under normal mitochondrial membrane potential, JC-1 forms red fluorescent aggregates, whereas under depolarized conditions it remains in the green fluorescent monomeric form.

Fluorescence intensities were measured using a microplate reader (red: Ex/Em = 535/590 nm; green: Ex/Em = 485/530 nm), and the red/green fluorescence ratio was calculated as an indicator of mitochondrial function. Fluorescence images were also captured for visualization.

### 4.9. Detection of Mitochondrial ROS and ATP

To evaluate mitochondrial oxidative damage and energy metabolism, mROS and ATP levels were measured. HUVECs were seeded into 24-well plates at a density of 4 × 10^4^ cells per well. For mROS detection, MitoSOX™ Red (MedChemExpress, Beijing, China) mitochondrial superoxide indicator was used. After treatment, cells were incubated with 5 μM MitoSOX™ Red at 37 °C for 15 min in the dark, followed by three washes with PBS. Fluorescence images were acquired using a confocal microscope (Ex/Em = 510/580 nm). Five random fields per group were analyzed, and fluorescence intensity was quantified using ImageJ.

ATP levels were measured using a luciferase-based assay. HUVECs were seeded into 6-well plates at a density of 4 × 10^5^ cells per well. After attachment, cells were treated with 1 μg/mL LPS (control group received PBS), followed by drug treatment (10 μg/mL) after 24 h for an additional 18 h. Cells were then lysed in 200 μL lysis buffer on ice for 10 min and centrifuged at 12,000 rpm at 4 °C for 10 min. The supernatant was collected, and ATP levels were measured according to the manufacturer’s instructions. Luminescence intensity was recorded using a microplate reader, and ATP content was calculated from a standard curve and normalized to protein concentration.

### 4.10. Endothelial Cell Migration Assay

Cell migration was evaluated using a wound healing assay. HUVECs in logarithmic growth phase were seeded into 6-well plates at a density of 5 × 10^5^ cells per well (three replicates per group) and cultured for 24 h until reaching ~90% confluence. Cells were then treated with medium containing 1 μg/mL LPS for 12 h to establish the inflammatory model (control group received PBS). A uniform scratch was created using a sterile 200 μL pipette tip, and detached cells were removed by gently washing with PBS three times. Cells were cultured in serum-free medium and treated with different formulations: PBS (control and model), Tan (10 μg/mL), Tan-CDs, AS-IV (10 μg/mL), and Tan-CDs@AS-IV.

Images were captured at 0, 6, 24, and 48 h using an inverted microscope, with three fixed fields per well. Wound area was measured using ImageJ, and migration rate was calculated as follows:

Migration rate (%) = (initial wound area − wound area at time t)/initial wound area × 100%

### 4.11. Tube Formation Assay

To evaluate the effects of drug treatments on the in vitro angiogenic capacity of endothelial cells, a Matrigel-based tube formation assay was performed. Prior to the experiment, Matrigel was thawed overnight at 4 °C, and all pipette tips and culture plates were pre-cooled to prevent premature gelation.

HUVECs were seeded into 6-well plates at 3 × 10^5^ cells per well and cultured for 24 h. Cells were treated with 1 μg/mL LPS for 12 h to establish the model, followed by treatment with different formulations (10 μg/mL) for 18 h. Cells were then harvested, resuspended in serum-free medium, and seeded onto Matrigel-coated 24-well plates (40 μL/well, pre-solidified at 37 °C for 30 min) at a density of 1 × 10^5^ cells per well. After 6 h of incubation, tube formation was observed under a microscope. Five random fields per well were captured, and angiogenesis parameters, including number of branch points, total tube length, and mesh number, were quantified using the Angiogenesis Analyzer plugin in ImageJ.

### 4.12. Live/Dead Cell Staining

Cell viability was further evaluated using Calcein-AM/PI dual staining. HUVECs were seeded into 24-well plates at 3 × 10^4^ cells per well and cultured for 24 h. Cells were then treated with 1 μg/mL LPS for 12 h to establish the injury model, followed by drug treatment for 24 h. After treatment, cells were washed three times with PBS and incubated with Calcein-AM/PI staining solution (final concentrations: Calcein-AM 2 μM, PI 4 μM) at 37 °C for 20 min in the dark. Cells were washed twice with PBS and imaged under a fluorescence microscope. Live cells exhibited green fluorescence, while dead cells showed red fluorescence. The percentage of viable cells was quantified using ImageJ.

### 4.13. Mitochondrial Transfer Assay

To evaluate intercellular mitochondrial transfer, a donor–recipient co-culture model was established. Donor HUVECs were seeded into 6-well plates at 4 × 10^5^ cells per well and treated with different formulations (10 μg/mL; model group with 1 μg/mL LPS, control with PBS) for 18 h. Recipient HUVECs were seeded at the same density and treated with 1 μg/mL LPS. Donor cells were labeled with MitoTracker Red CMXRos (Beyotime Biotechnology, Shanghai, China) (200 nM, 30 min, 37 °C), while recipient cells were labeled with Calcein-AM (Beyotime Biotechnology, Shanghai, China) (2 μM, 30 min). After staining, both cell types were washed and mixed at a 1:1 ratio, then seeded into confocal dishes at 2 × 10^4^ cells per dish and co-cultured for 4 h. Cells were stained with Hoechst and imaged using a confocal microscope. The proportion of recipient cells containing transferred mitochondria was quantified.

### 4.14. Western Blot Analysis

Protein expression was analyzed by Western blot. HUVECs were seeded into 6 cm dishes at a density of 1 × 10^6^ cells per dish. After 24 h, LPS-induced injury and drug treatments were applied for 24 h. Cells were washed with cold PBS and lysed with RIPA buffer containing protease and phosphatase inhibitors (200 μL per dish) on ice for 30 min. Lysates were collected, centrifuged at 12,000 rpm at 4 °C for 15 min, and the supernatant was obtained. Protein concentration was determined using a BCA assay. Samples were mixed with loading buffer, denatured at 95 °C for 5 min, and separated by 10% SDS-PAGE, followed by transfer onto PVDF membranes. Membranes were blocked with 5% skim milk for 2 h at room temperature and incubated overnight at 4 °C with primary antibodies against PGC-1α and Cx43 (1:1000). After washing with TBST, membranes were incubated with HRP-conjugated secondary antibodies for 2 h at room temperature. Protein bands were visualized using ECL reagents and imaged with a gel imaging system. Band intensities were quantified using ImageJ.

## Data Availability

The data that support the findings of this study are available from the corresponding author upon reasonable request.
